# What Prevents Quality Midwifery Care? A Systematic Mapping of Barriers in Low and Middle Income Countries from the Provider Perspective

**DOI:** 10.1371/journal.pone.0153391

**Published:** 2016-05-02

**Authors:** Alex Filby, Fran McConville, Anayda Portela

**Affiliations:** 1 National Health Service of England and Wales, The Whittington Hospital, London, United Kingdom; 2 Department of Maternal, Newborn, Child and Adolescent Health, World Health Organization, Geneva, Switzerland; University of South Australia, AUSTRALIA

## Abstract

**Background:**

Quality of care is essential for further progress in reducing maternal and newborn deaths. The integration of educated, trained, regulated and licensed midwives into the health system is associated with improved quality of care and sustained decreases in maternal and newborn mortality. To date, research on barriers to quality of care for women and newborns has not given due attention to the care provider’s perspective. This paper addresses this gap by presenting the findings of a systematic mapping of the literature of the social, economic and professional barriers preventing midwifery personnel in low and middle income countries (LMICs) from providing quality of care.

**Methods and Findings:**

A systematic search of five electronic databases for literature published between January 1990 and August 2013. Eligible items included published and unpublished items in all languages. Items were screened against inclusion and exclusion criteria, yielding 82 items from 34 countries. 44% discussed countries or regions in Africa, 38% in Asia, and 5% in the Americas. Nearly half the articles were published since 2011. Data was extracted and presented in a narrative synthesis and tables. Items were organized into three categories; social; economic and professional barriers, based on an analytical framework. Barriers connected to the socially and culturally constructed context of childbirth, although least reported, appear instrumental in preventing quality midwifery care.

**Conclusions:**

Significant social and cultural, economic and professional barriers can prevent the provision of quality midwifery care in LMICs. An analytical framework is proposed to show how the overlaps between the barriers reinforce each other, and that they arise from gender inequality. Links are made between burn out and moral distress, caused by the barriers, and poor quality care. Ongoing mechanisms to improve quality care will need to address the barriers from the midwifery provider perspective, as well as the underlying gender inequality.

## Introduction

Global research has concluded that midwifery care has a pivotal role in the reduction of preventable maternal and newborn mortality and morbidity [1]. The increased access to skilled attendance at birth in the low and middle income countries (LMICs) that contribute to 99% of the global maternal mortality rate, has not, however, resulted in expected reductions in mortality [2]. This can be explained by a lack of quality maternity care [3, 4]. The evidence indicates that strengthening midwifery is key to improving quality of care and achieving international efforts; yet implementation of educated, trained, regulated and licensed midwives remains inconsistent, resulting in a critical obstacle to progress [5].

The “three delays” model [1994] identified barriers to accessing care from the perspective of childbearing women; (1) delay in the decision to seek care; (2) delay in arriving at a health facility and (3) delay in the provision of adequate care at the facility [6]. The perspective of the women who provide that care, however, has remained virtually absent from the discourse [7]. To initiate the discussions on the possible barriers experienced by midwifery personnel in providing care, the World Health Organization (WHO), in collaboration with the International Confederation of Midwives (ICM) and the White Ribbon Alliance (WRA), convened a session at the *2013 Women Deliver Conference* to determine if providers of midwifery care felt *empowered*, *respected* and *safe* [8, 9]. The research presented by delegates from Nepal, Papua Guinea and Afghanistan and the ensuing multi-country discussions, highlighted the shortcomings in the education, training, licensure and regulation of professionals, while also detailing the significant personal challenges that women who provide midwifery care face [10]. This includes: social inequality, inadequate pay to meet the basic cost of living, unsafe working conditions and physical and sexual abuse [9]. The negative impact of these realities on quality of care was described through the concepts of *burn out* and *moral distress*. *Burn out* is defined as the expenditure of energy, effort and time on work without adequate time or environment to recover physically and emotionally [11]. Moral distress is defined as the experience of being seriously compromised as a moral agent by being unable to practice in accordance with accepted professional values and standards. This is associated with frustration, anger, guilt, anxiety, perceived lack of control, feeling belittled or unintelligent, and negative physical symptoms [12].

**Fig 1:**
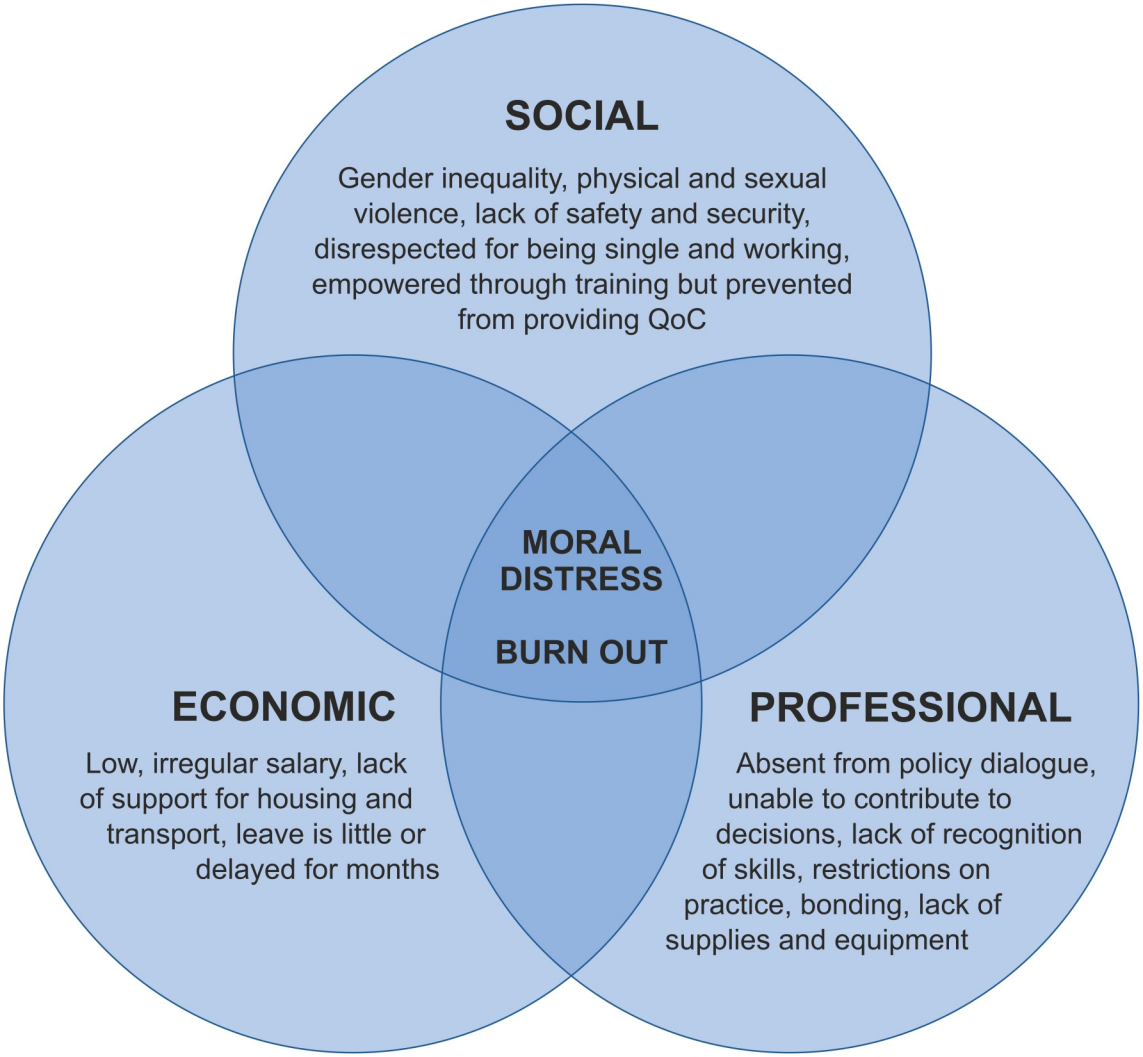
Analytical framework: barriers to the provision of quality of care by midwifery personnel.

Analysis of the findings from the *Women Deliver* session enabled categorisation of the multiple issues faced by the women who provide midwifery care into social, economic and professional barriers, and resulted in the development of an analytical framework (Fig 1). This framework presents burn out and moral distress as a consequence of interactions between all three barriers.

It has recently been recognised that the complexity of access to quality of care goes beyond a health and development issue and requires a broader human rights approach [13], thinking beyond the practicalities of health systems to include human relationships, desires and values, roles and norms, and power structures [14]. Maternal and newborn mortality reduction is described as being hampered by gender inequality on two fronts—the gender discrimination experienced by the woman who provides the care and the additional gender inequality experienced by the childbearing woman [15]. Midwifery is unique within healthcare, being represented nearly exclusively by women and traversing both domestic and medical domains and cultures [16]. The recently released Global Strategy for Women’s, Children’s and Adolescent Health (2016–2020) highlights the need for further progress to be based on gender responsive, equity driven and rights based approaches [17].

### Aims and objectives

We conducted a systematic mapping of the literature to describe the literature that answers the question: *What are the social*, *economic and professional barriers preventing midwifery personnel in low and middle income countries (LMICs) from providing quality of care to mothers and newborns*?

The objectives were to develop a map of the literature on barriers to quality midwifery care through a methodical and replicable process and establish the relevance of the analytical framework developed through the *Women Deliver* session by detailing the barriers found and the type of literature identified.

## Methods

A systematic mapping allows materials from a range of sources to be identified and does not exclude items based on study design or literature type, while still providing a process that is methodical and replicable [18]. This approach is particularly helpful in identifying gaps for further reviews and primary research for topics where it is anticipated that effectiveness studies will not be found which can support specific outcome-focused questions. We developed a protocol, which is available from the corresponding author.

An area of anticipated difficulty for the mapping was the definition of midwifery personnel. Midwifery has been described as “commonly misunderstood” [5] with midwifery care providers lacking a universally protected and acknowledged title. We reviewed different definitions [5, 19, 20]. In order to focus on the wider range of professional groups who are, in many circumstances, providing elements of midwifery care, we adopted the WHO/ICM/FIGO definition of a skilled birth attendant (SBA) to represent midwifery personnel:

*An accredited health professional—such as a midwife*, *doctor or nurse—who has been educated and trained to proficiency in the skills needed to manage normal (uncomplicated) pregnancies*, *childbirth and the immediate postnatal period*, *and in the identification*, *management and referral of complications in women and newborns*. [20]

Although this definition includes doctors, the authors felt that medical professionals are not subject to the same social and economic inequality and misunderstanding over professional title and status as are other midwifery personnel and therefore as indicated further below, medical professionals were not considered in this mapping.

The search was conducted on the 20^th^ August 2013. The intervening time between the search and article submission was used to analyze the data and draft the paper for submission.

### Inclusion/exclusion criteria

All eligible items that discussed barriers to midwifery personnel providing quality care were considered, including published and unpublished material, whether in print or online such as journal articles, news items and project reports from governments and other agencies in all languages. Since the aim is to describe the literature available for the question framed above, items were not assessed for quality or excluded based upon study design. The classification of barriers as social, economic and professional was applied in the analysis stage and did not affect inclusion of articles during the screening process.

Items from LMICs, classified according to the World Bank criteria [21] were included. This was justified in order to focus on quality of care in the countries that contribute to 99% of the global maternal mortality rate [2].

Midwifery personnel who met the definition of an SBA as defined above, regardless of professional title were included. Items exclusively discussing medical professionals, including obstetricians and gynaecologists were excluded. Items, however, that discussed obstetricians and gynaecologists collectively with other midwifery personnel were considered, so as not to discard relevant material. The length and content of the professional training was not considered, as long as it resulted in SBA status.

Items that focused on traditional birth attendants were excluded. Items from high income countries and published before January 1990 and after August 2013 were also excluded.

### Search strategy

We developed an initial search strategy based on the contributions received from a call for papers from WHO regional offices, midwifery associations, advocacy groups and individual experts. Five relevant references were received, all of which were published items, and were used to pilot electronic searches on Pubmed and CINAHL. The terms developed for barriers were the most challenging aspect of the search design and were largely informed by the analytical framework (Fig 1) and *The State of the Worlds’ Midwifery Report 2011* [2]. The initial piloting process also developed additional terms such as ‘lived experience’, ‘voice’, and ‘opinion’ which returned many relevant items. The LMIC criteria were met by adding a pre-designed filter developed by the WHO.

A systematic search of five bibliographic databases was then conducted. The databases selected were PubMed and The Global Index Medicus, for breadth; The Maternity and Infant Care Index and CINAHL, for a nursing and midwifery focus; and POPLINE, for a reproductive health focus. This selection was intended to capture relevant literature across the disciplines of midwifery, nursing, medicine, social science, health systems, and health policy.

The search strategy was specifically adapted to each electronic database and Medical Subject Headings (MeSH) used where possible. Midwifery personnel terms were searched in combination with approximately 150 terms for ‘barriers’ and a LMIC country filter applied. The search terms are detailed in the Supporting Information (S1 Table) accompanying this article.

**Fig 2:**
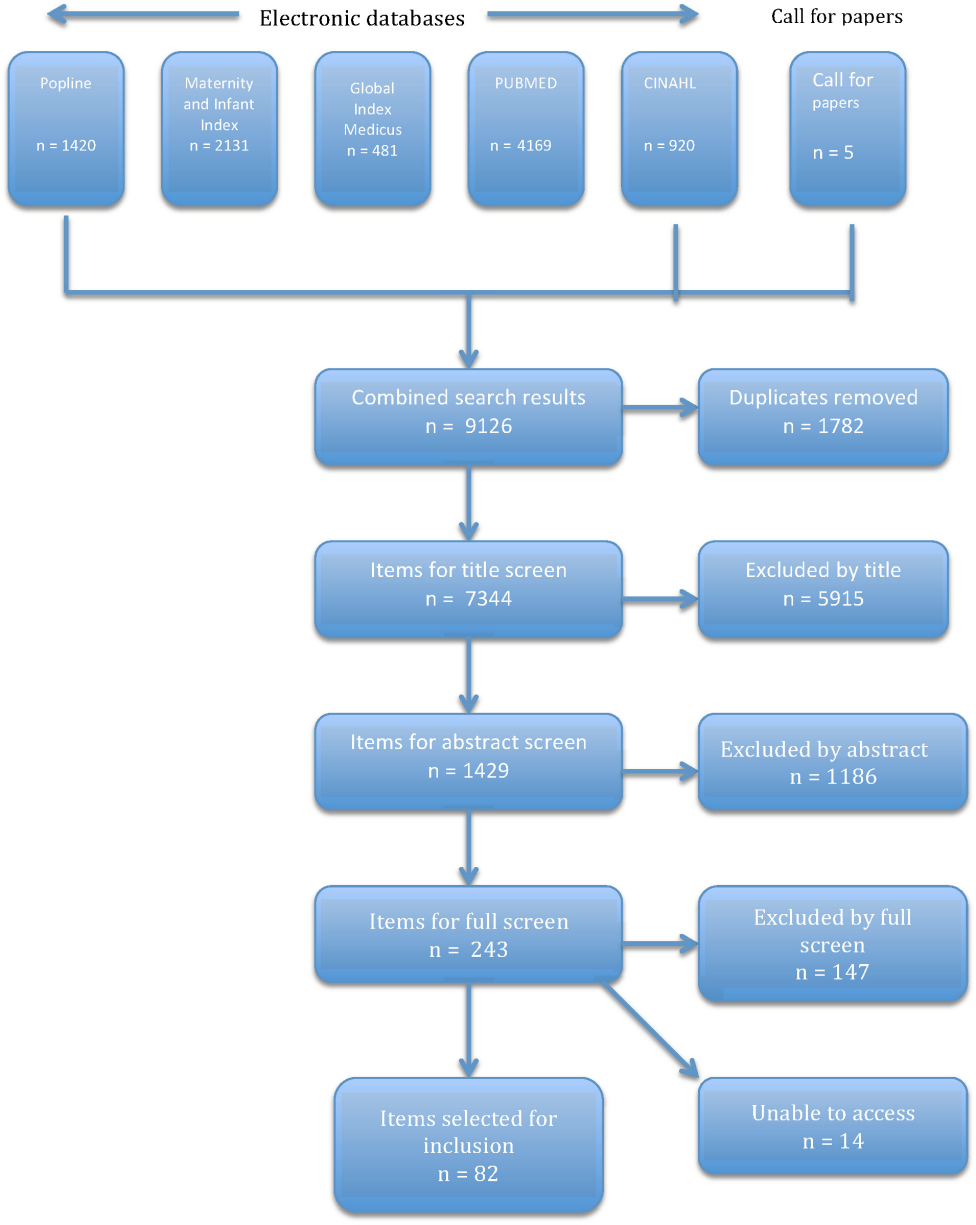
Mapping results.

The search yielded 9126 items across the five databases and the call for papers, which was reduced to 7344 items, once duplicates had been removed. Two of the authors (AF and FM), screened the articles by title and the total was reduced to 1429 items. When a decision could not be reached using the title alone, the item was included for abstract review and full text access as necessary. Two of the authors (AF and FM) reviewed the remaining abstracts independently and met to discuss any discrepancies in judgement. A total of 243 items were selected for full-text access. Two of these items were books, which were also accessed and screened. Of these 243 items, 14 could not be accessed. Items were reviewed by two authors (AF and FM) and discussions were held regarding items that presented difficulty. 147 items failed to meet the inclusion criteria; with a final total of 82 articles included. This screening process is represented in Fig 2.

## Findings

Data was extracted for analysis from all 82 items including: literature type, year of publication, country of origin, midwifery professional title and category of barrier as per the analytical framework (Fig 1). All included items are listed in the Supporting Information (S2 Table) accompanying this article.

More than half (60%) of the selected items were published research journal articles. These were all descriptive studies, except for one intervention study. In addition, the mapping identified commentaries without methods (32%), international agency reports (5%), a news item (1%), a press release (1%) and a letter (1%). Only one foreign language item was identified (French) for which an English translation was available.

The terminology adopted for midwifery personnel was *midwives* (60%), followed by *maternity staff* (17%) which included midwives with other personnel, (e.g. obstetricians, paediatricians, physicians and neonatal nurses) *nurse-midwives/nurses* (12%) or *SBA*s (8%). Only 3.6% of the findings referred to additional cadres who had SBA status, for example, Community Health Extension Workers (CHEWs) in Nigeria.

Of the 82 included items, 71 (87%) were published either in or after 2005, and 34 of these (42% of the total number of items) were published since 2011. Of the included items, 44% discussed countries or regions in Africa, with Ghana, Uganda and South Africa being the most common of these; 38% discussed Asian countries or regions, with Indonesia, Afghanistan, Nepal and India being the most common; only 5% discussed barriers in the Americas region; 13% covered LMICs as a general group rather than specific countries; 1% discussed LMICs within a global context. Countries were classified into regions according to the *United Nations Classification of Countries by Major Region and Area of the World* [22].

Most items (93%) fell into the category of professional barriers, followed by economic (42%) and then social (38%). Below we describe the different items in each category, while acknowledging the interaction between categories and clarifying this where possible.

### Social barriers

We identified 31 items describing social barriers to the provision of quality midwifery care. We found that many of the social barriers had a strong underlying link to the socially and culturally-constructed context of childbirth as well as gender inequality.

A global policy guidance report concluded that gender inequality and lack of female empowerment was the most significant barrier to the advancement of the midwifery profession [23]. This assertion was echoed at a national meeting of midwives in Afghanistan [24]. Interviews with midwives in Anambra state, Nigeria found that deeply embedded gender inequalities predetermine the low social status of the midwifery profession [25]. This will be further explored in the section below under professional barriers and is associated with professional disrespect and a perceived lack of authority by midwifery personnel [23, 24], and in some instances a lack of government commitment [26].

A global report and items concerning Zimbabwe and Afghanistan, suggested that cultural influences construct a perception of assisting childbirth as low skilled and inherently ‘women’s work’ [24, 26]. The association between the low social status of women and attending births was generic, yet culturally specific in its manifestation. For example, research from South Asia described how some Hindu and Muslim families construct a temporary, separate structure for birthing in a *dirty* area [27]. Relatives and neighbours may watch and question the attendant's work yet refuse to provide assistance (including a drink of water) for fear of contact with *polluted* bodily fluids associated with menstruation, childbirth and colostrum [27]. Research from Pakistan found that women who provide midwifery care were described as “uneducated women of doubtful moral character”, and therefore an unsuitable role for a respectable Muslim woman [28].

The promotion of evidence-based care by midwifery personnel can be constrained by social barriers. In Mozambique, for example, midwifery personnel hesitated to promote evidence-based skin to skin care at birth. This was due to societal attitudes that the newborn requires cleansing prior to contact, as the childbearing mother’s blood is considered unclean[29, 30]. A study from Ghana found that 70% of mothers ignored professional advice regarding care of the umbilical cord, deferring instead to their grandmothers’ guidance [31]. In Bangladesh, procedures based on best practice may require the consent of the older women in the family [27]. In Angola efforts by midwifery personnel to increase health facility births were met with resistance due to facility-based practices that do not reflect cultural norms, with only homebirth being acceptable [32]. Generally across low and middle income countries, and specifically, Indonesia, TBAs were preferred over midwifery personnel as they were seen by women and communities as trustworthy due to their respect for religious beliefs and cultural practices [33, 34]. Midwives in Niger and Iran could face social and cultural barriers when providing information about sexual health and contraception in the presence of men, and could be culturally forbidden from using terms related to sexuality [35, 36].

Social isolation was reported by midwifery personnel in different contexts [29, 32, 35, 37–40]. Midwifery personnel in rural Nigeria, Niger and Ghana, were typically young and single and rarely had a social connection with the community to which they were deployed. They were often not accepted by the community due to their age, and had little opportunity for marriage or starting a family [35, 37, 38]. In studies from Afghanistan and Burkina Faso, midwives cited not speaking the local language as another reason for social isolation from their assigned communities [39, 40].

In some contexts where midwifery has been professionalized, midwifery personnel experienced prejudice for being regarded as too educated and transgressing traditional gender roles [39, 41, 42]. An example from Mali noted that in communities where midwifery personnel were the only educated and salaried females, there can be feelings of jealousy and resentment from local women [42]. An example from Afghanistan revealed how the professionalization of midwifery could be politically sensitive: the new professional Afghani midwives fulfilled a traditional role, yet also represented educated, independent women [39]. In a culture where it is generally unacceptable for young women to live away from home for study or work, community midwives were reliant on their families granting them permission to work [39]. Midwives had to be accompanied by a male relative, and clinics were guarded by security at night due to threats to the midwives’ safety, with one example of a clinic being set on fire [22, 39, 41].

In some contexts, midwifery personnel were extremely vulnerable when attending homes or leaving work late at night, with Ugandan and South African midwives reporting physical attacks [43, 44]. In Bangladesh, despite being accompanied by a porter for night calls, female mobility of midwifery personnel after dark was associated with inviting sexual assault [27]. Young, unmarried women who provide midwifery care, living in rural areas without secure accommodation were concerned for their safety [43, 45] and some were unable to provide 24-hour quality care due to the risk of sexual harassment and violence [43]. This was again linked to the low social status of community midwives underlying the lack of investment in secure accommodation and safe travel to support their services [39]. Where access to midwifery care is compromised so is quality of care for women and newborns.

Midwifery personnel across LMICs discussed the expectation to fulfil their unpaid domestic and reproductive roles alongside their professional one [26, 45, 46, 47]. The specific demands of the midwifery role, with excessive working hours outside the home, especially at night, lead to consequent suspicions of infidelity and spousal abandonment [30, 47]. A study of midwifery personnel in Malawi suggested that domestic duties, child rearing and accountability to one’s spouse, possibly with a lack of spousal support, could negatively affect job performance and result in a sense of depersonalization and professional inadequacy [48].

### Economic barriers

Economic barriers to the provision of quality of care were described in 34 of the 82 items. Economic issues included low or absent wages, informal payments and a lack of governmental financial commitment. The low wages and economic difficulties described link to the findings below that portray midwifery as an unvalued profession, which in turn refers back to the low social status discussed above.

Items from Afghanistan, Indonesia, Anambra state; Nigeria, Uganda, Koutiala; Mali, as well as Africa-wide and LMICs collectively [25, 30, 42, 47, 49, 50, 51, 52] revealed that many midwives were surviving on wages which fail to meet basic living costs, with salaries paid infrequently, or not at all. Midwives in Angola reported that they were often paid three to six months in arrears, and Afghan midwifery personnel indicated they can wait up to six months for their public salary [32, 39]. In Mali, on-the-spot fees from women were redistributed as salaries by the local community health committee at unpredictable intervals and amounts [42]. Midwives in three different maternity units across Luanda, Angola, reported that salaries were too low to provide food, clothes and education for their children [32]. This was echoed by a global commentary that also noted that wages were inadequate to purchase essential protective equipment, such as gloves, which midwifery personnel were required to provide from their domestic income, if their employer failed to [49].

Midwifery personnel in several African nations and in Indonesia were required to charge obligatory user fees, or be reimbursed by fee exemption schemes, for their remuneration. This difficult and unreliable process poses another economic barrier. It can also pose a social barrier: The assumption that midwifery personnel will require a cash payment for their services can foster distrust and resentment towards them by the local community and reinforce lack of acceptance [30, 41, 51, 52]. In Indonesia, midwifery personnel were not always reimbursed for attending women entitled to an official exemption fee and had to prioritize giving care to women that could pay them directly [51]. In Burkina Faso, midwifery personnel indicated that quality of care could be effected by the financial stress incurred when they had to take out loans to support themselves [40]. Just to survive, some midwifery personnel had to take on additional, non-midwifery employment [46, 53].

For village midwives in Indonesia, and nurse–midwives in Peru, the earning potential of moving to a city outweighed any rural government subsidy and therefore contributed to low rural retention levels [52, 54]. In Senegal, midwifery personnel linked their inadequate remuneration with low motivation, low self-esteem and low job satisfaction [53].

### Professional barriers

Professional barriers were identified in 76 of the 82 items. Professional barriers could be grouped into: a lack of investment in quality midwifery education; weak or absent regulation; inadequate numbers of staff; lack of affordable transport; weak facility management and poor working conditions. Whilst the issues described here are not necessarily unique to midwifery care providers, they were found to be intensified due to the low socio-cultural status of the profession and gender inequality. This reflects midwifery personnel largely being women, who are exclusively concerned with women and childbirth.

Midwives interviewed in seven cities across China identified a lack of investment in quality midwifery education and training as one of the most significant barriers to the advancement of the midwifery profession[55]. Research from Afghanistan, Bangladesh, Bhutan, India, Nepal and Pakistan, China, and Africa in general, concluded that inadequate training was considered to jeopardize professional identity, competence and confidence of midwives as the primary care giver for maternal and newborn health [30, 41, 55, 56, 57, 58].

It is suggested that short-term, unregulated “SBA” courses, ranging from six weeks to one year in length, detracted investments away from midwifery training to international standards [25, 56]. While coverage of SBAs was measured through the global Millennium Development Goal 5 indicator, the quality of care provided was not quantified [26]. Short courses across LMICs and, in South Africa specifically, were reported to underestimate the level of decision making and responsibility required to manage and refer women who need emergency obstetric interventions [58, 59]. This was especially noted for those working alone in remote areas without medical support [27, 58, 57]. Short training of multi-purpose health workers was said to also have a negative impact on the quality of care for women and newborns and was considered to be neither cost effective nor sustainable [23, 25, 26, 60, 61].

A continuing barrier to improving midwifery training included the lack of investment in faculty who were competent in education, theory and clinical practice [26, 58, 62]. In Jordan, midwifery was commonly being taught by nurse educators or doctors with little knowledge of the midwifery model of care [63]. Lecturers across LMICs and more specifically, some African countries, were reported to be often disconnected from the clinical areas, with no resources for updating their clinical knowledge, causing students to graduate with outdated practices [23, 26, 30, 58]. In some countries in Africa, training was typically aimed at highly resourced urban and institutionalized care with medical support, with little adaptation to the national context [30, 58], and yet newly qualified midwifery personnel were often deployed to rural posts without prior community practice experience, supervision or support in dealing with emergency situations [30, 58, 60, 64].

**Fig 3:**
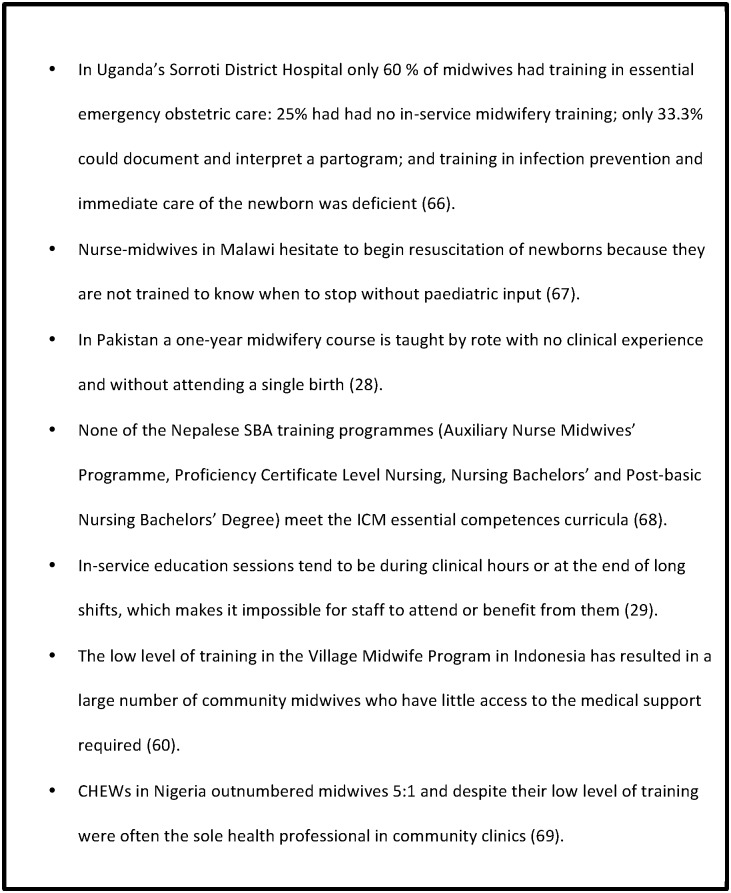
Examples of how lack of investment in midwifery training prevents quality midwifery care.

In Afghanistan, poor quality training was found to impact on midwifery personnel through discrimination by other providers, especially doctors, who ridicule their ability [41]. The strength of the medical hierarchy caused midwifery personnel to defer clinical decision making to inexperienced junior doctors [29, 30, 65], yet midwives in Mozambique noted that at night they were expected to manage care without resource to medical teams [29]. Examples from the literature of how lack of investment in midwifery training prevents quality midwifery care are presented in Fig 3.

Lack of investment in certified registration, effective regulatory bodies or professional associations means that regulatory bodies were unable to enforce the licenses that ensure quality in training and practice [25, 68]. In Nepal, this led to midwifery training and professional titles for midwifery practitioners to become diverse and non-indicative of skill level [68]. Shortfalls in training and poor clinical practice were rarely addressed and midwifery personnel have little support for accountability in their practice and little evidence of professional development [39, 55, 56, 58].

**Fig 4:**
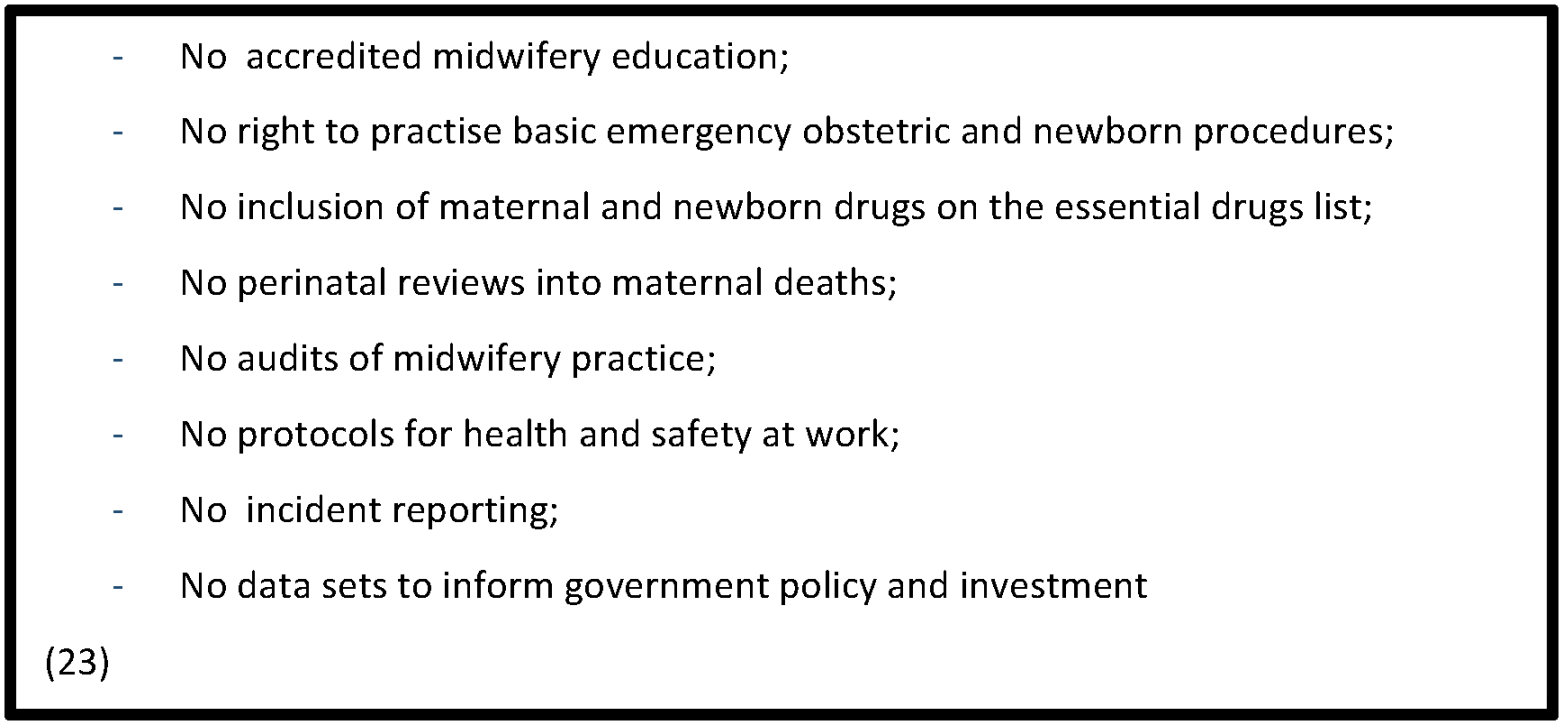
How absence of regulatory frameworks and professional associations prevents quality midwifery care.

Where there were midwifery associations, for example in Zimbabwe, members were described as lacking the necessary experience and skills required for leadership and management, as well as basic office facilities [70]. In Zimbabwe, the finances of the association were found to be completely dependent on membership fees which, due to the low salaries and emigration of midwives, leaves little funding for activities or strategic planning [70]. In Gujarat, India, where midwifery was found to be represented by a nursing regulatory body, midwifery personnel were neither recognised as autonomous professionals nor deployed as midwives [71]. Articles from India, Jordan and LMICs generally, suggested that the integration of midwifery and nursing could make the midwifery profession a subsidiary to nursing. This could deny it a unique professional identity and voice and discourage the emergence of strong midwifery leaders [57, 58, 63, 71]. A study from Anambra State, Nigeria concluded that the lack of a strong unionized voice for midwives diminished the profession’s recognition and reinforced the implementation of programmes for low skilled multipurpose workers [25]. Fig 4 provides a summary of how the absence of regulatory frameworks and professional associations prevents quality midwifery care.

**Fig 5:**
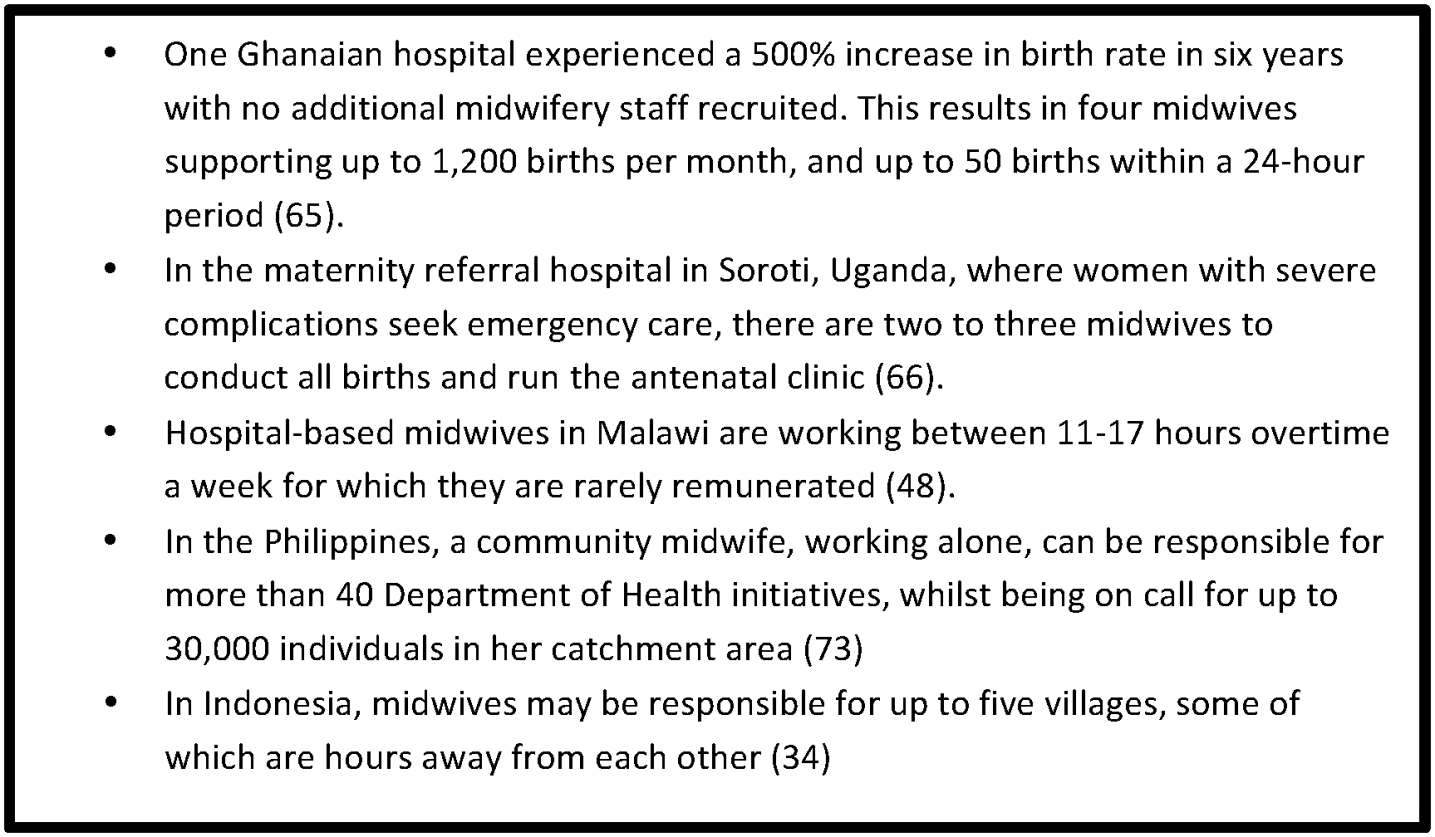
Examples of how heavy workload prevents quality midwifery care.

Inadequate staffing levels and an increasing workload was an issue across both urban and rural settings [50, 58, 65]. A report concerning African countries stated that inadequate staffing and working excessive overtime was found to compromise safety for women, as well as midwifery personnel [30]. The impossible demands of their workload could place midwifery personnel in an ethical dilemma of how to prioritize care: Nurse-midwives in Malawi spoke of the daily problem of having to decide whether to care for the newborn or the mother, or even, another mother and another newborn [67]. In Uganda, the breadth of the midwifery role has been extended beyond care that can be provided with quality, with health centre midwives caring for 50–60 women per day including providing all immunisations, family planning, HIV counselling, as well as, antenatal care and supporting women during childbirth [47]. The psychological impact of an overwhelming workload and being forced to neglect those under their care was associated with significant low morale, burn out and moral distress [50, 66, 72]. Fig 5 presents examples of how heavy workload prevents quality midwifery care.

Quality of care can be further compromised by poor working conditions and insufficient basic resources, including scarcity of water, sanitation, drugs and equipment [25, 30, 45, 47, 65]. Midwives in rural Uganda described using mobile phones held in their mouths as a light at births because the electricity had been cut off during an attack eight years earlier [74].

The absence of safe working conditions, such as sharps disposal, water for hand washing, and basic protective supplies such as gloves, as well as limited access to Post Exposure Prophylaxis (PEP) had left midwives highly vulnerable to HIV infection in the workplace [45, 72, 75]. Midwives in one region of Zambia, forty percent of whom were HIV positive, reported withholding information about their infection status and accidents such as needle stick injuries, for fear of stigma or losing their jobs [30].

Inefficient or absent transport with impassable or dangerous roads was also a recurrent barrier to providing quality midwifery care in both urban and remote areas [38, 40, 51]. Midwifery personnel attended women on foot, by rickshaw, bicycle or horse, carrying minimal supplies through monsoons and floods, and often arrived too late [27, 73]. In Nepal, it was estimated that SBAs were able to provide antenatal care to only 7.2% of pregnant women in the hill and mountain districts and attend 1.4% of the expected births within their area [76]. During the dry season in Bangladesh, midwifery personnel were unable to visit women as they relied on the monsoon season boats for transport [27]. Night calls were particularly difficult and dangerous to attend, and routine home visits were not possible [27]. carabao see bubalus bubalis.

### Burn out and moral distress

The analytical framework (Fig 1) theorized that the interaction of social, economic and professional barriers resulted in moral distress and burn out. Whilst the expression ‘moral distress’ was not adopted by providers, they did express corresponding feelings of guilt, anger, depersonalization and demoralization that fit the definition [29, 40, 45, 53, 72]. This was predominately caused by feelings of inadequacy in the face of an overwhelming maternal and newborn mortality rate [30, 32]; not being able to provide best practice or lacking skills to work autonomously [29, 36, 65, 77, 78]; and being required to manage complications beyond their competency [27, 30]. The term *Burn out* appeared in the literature to describe the impact of conditions upon midwifery personnel and was associated principally with exhaustion and frustration [26, 48, 50, 53, 72]. A study of Malawian midwives suggested that they were at higher risk of burn out than other clinicians [48]. A global report suggested that the level of burn out amongst midwifery personnel may be due to the predominance of women in the profession and the resulting tension between their professional role and domestic lives [26]. This is an example of how social barriers interact with professional barriers, with the pressure to fulfill their domestic role as a woman increasing the personal impact of excessive working hours.

### Limitations

One particular challenge was to map the concept of *barriers to the provision of quality midwifery care*. While this was informed by the analytical framework (Fig 1) and *The State of the World’s Midwifery Report 2011* [2], these sources were not exhaustive and some relevant terms may have been omitted. Test searches were used to establish additional terms. This method, while useful, depended upon the authors’ ability to suggest relevant terms to test and was therefore open to omissions. Identification of relevant items and the assignation to a category was discussed at length between the authors but remains a subjective process.

We did not identify any grey literature through our search methods; there is a probability that relevant material has been missed. In addition, 14 items could not be accessed for full text screening and may have been eligible.

Finally, we note that the search concluded in August 2013 due to finite resources. Nonetheless we feel the literature gathered reached the objective of validating the components of the framework.

## Discussion

This first systematic mapping of the literature on barriers to quality midwifery care in LMICs has identified a variety of sources and has established a commonality of barriers. It has consolidated the contributions from the Women Deliver 2013 meeting and explored the relevance of the categories of social, economic and professional barriers [8].

The social barriers, despite being the least reported in the mapping, were found to be significant in preventing quality midwifery care. As described above, we found that many of the social barriers had a strong underlying link to the socially and culturally-constructed context of childbirth in which midwifery personnel work. These barriers are attributed to childbirth being historically the domain of the home and the responsibility of women [16]. Midwifery care is therefore bound with the domestic and the feminine.

The concept of women’s triple roles provides an explanation: *reproductive* (childbearing), *productive* (economic), and *community managing* (e.g. unpaid work in support of the community) [79]. In contrast to the mainly productive role of men, societies expect women to simultaneously undertake their triple roles with neither additional remuneration nor improved social status [79]. Midwifery personnel not only face this triple responsibility as women, but uniquely face having their *productive* role as care providers culturally perceived as belonging to the *reproductive* (childbearing) context, which confuses and undervalues their economic and professional contribution to society [79]. Additionally, the literature highlights that, in some contexts there is vulnerability to physical and sexual assault when providing care [27, 41, 44], as well as a lack of societal acceptance of trained midwifery personnel [41, 42]. A lack of acceptance, especially in societies where the socio-cultural barriers dominate, is likely to limit investment in quality midwifery care.

Economic issues were the second most common barrier discussed in the selected items and included: low or absent wages, the need for additional non-midwifery employment, informal payments and a lack of governmental financial commitment. Although many health workers experience professional and economic barriers, professions disproportionately comprised of women are described as experiencing a “gender penalty” [15], with men assuming the leadership position and women falling to the bottom of the occupational hierarchy and subsequently earning lower wages, largely because the job related skills are not treated as skills, but qualities of “being a woman” [15]. For midwifery personnel these professional and economic barriers are reinforced and intensified when they interact with the uniquely feminized profession of midwifery [23–25, 26, 47]. The United Nations Research Institute for Social Development states that when care is decently paid and protected, the interest of both the providers and users of care can be met, and has far reaching implications for gender relations and inequalities [80].

The low status of midwifery personnel, determined by gender inequality, contributes to the lack of financial and political commitment to investing in education, training, regulation and licensing [23 26, 63]. This also extends to inadequate investment in secure accommodation, transport and essential resources to enable midwifery personnel to perform their full role [43, 45]. These economic barriers reflect and reinforce the socio-cultural and professional perceptions of midwifery as low skilled and domestic work.

Professional barriers were the most frequently discussed, and revealed a lack of shared understanding about what midwifery is, and the level of education, training, support and regulation that is required to enable women and newborns to receive quality midwifery care [5]. This can extend to a lack of shared political understanding of the role of midwifery, and further limit investment in quality services [25, 26]. Professionally, this can be intensified where midwifery is considered as a subsidiary of nursing, preventing a collective midwifery voice and effective leadership [58, 63, 71]. This lack of understanding of midwifery may explain the perception of the role as essentially “women’s work” [24, 26] and why gender inequality is described as the primary barrier to the advancement of the midwifery profession [25].

The authors suggest that moral distress and burn out result in midwifery personnel being disempowered to provide quality of care [29, 45, 50, 78, 79, 81]. This finding is consistent with other research, which has shown burn out to affect the quality of services and patient outcomes through the adoption of negative and unprofessional behaviors [82]. While there is a potential link with this to the mistreatment of women during childbirth [83], we are unable to establish this here. The long term impact of burn out and moral distress is poor retention of maternity staff [30, 40, 50]. This outcome worsens the problems facing midwifery personnel, and increases pressure on those that remain [30].

**Fig 6:**
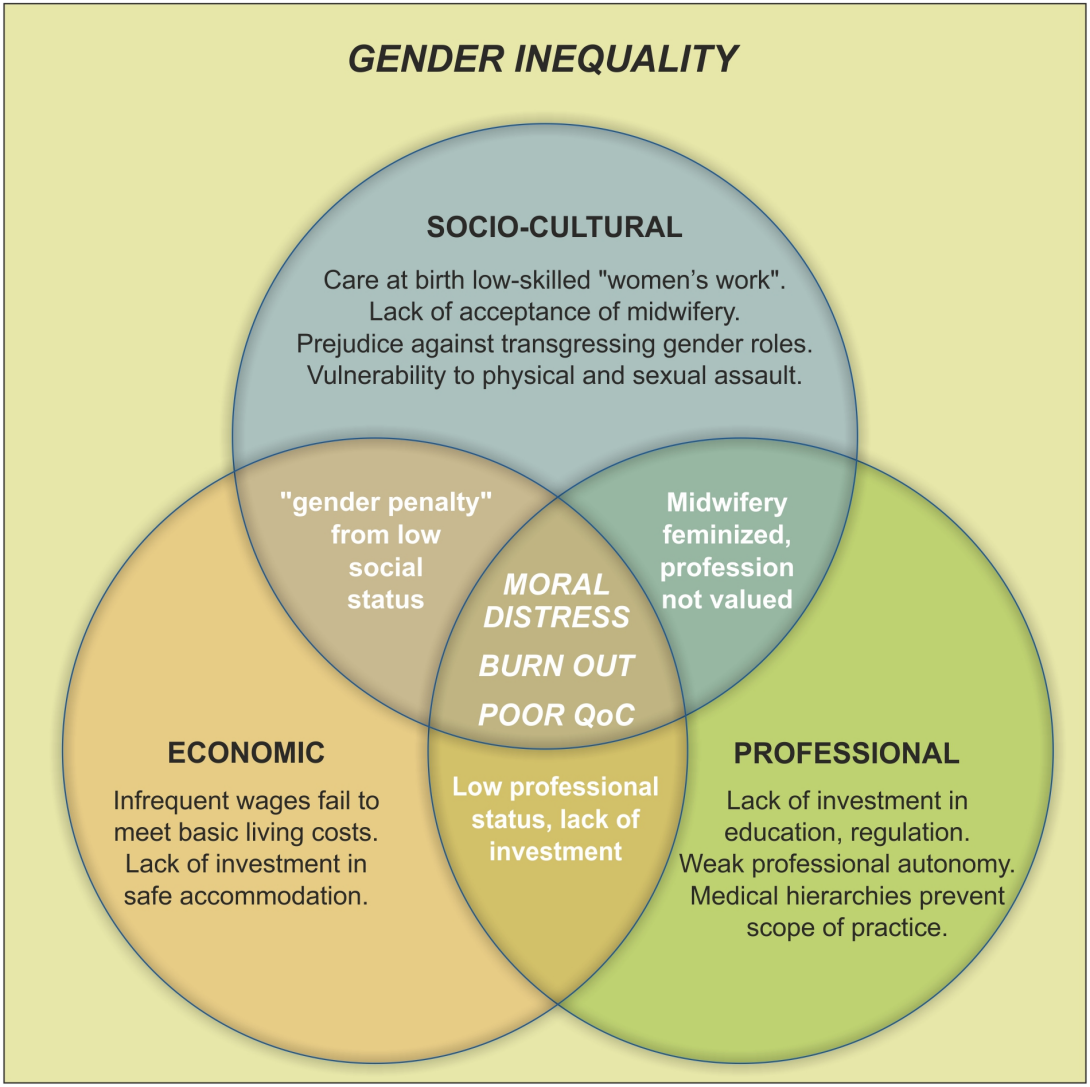
The revised analytical framework for barriers to the provision of quality of care by midwifery personnel.

The findings reveal enhancements that can be made to the original analytical framework, and we propose a revised version (Fig 6). A first enhancement is the adaptation of the social barriers to include cultural barriers, as these are entwined and specific to childbirth. The “social” category is therefore changed to “socio-cultural”. Secondly, the literature mapping revealed the dynamic between the barriers, which reinforce one another. Thirdly, gender inequality is a basis for all three barriers. Lastly, the complex interaction of the barriers along with gender inequality can result in moral distress and burn out and can, consequently, lead to poor quality of care.

## Conclusion

Global strategies to reduce maternal and newborn mortality and morbidity are placing increasing emphasis on quality of care [1]. Midwifery, provided by educated, trained, regulated, licensed midwives is associated with improved quality of care and rapid and sustained reductions in maternal and newborn mortality [84]. The findings of this mapping suggest, however, that the provision of quality midwifery care can be prevented by socio-cultural, economic and professional barriers, situated in gender inequality (Fig 6). This can be explained by the low socio-cultural status of midwifery—seen as “women’s work” [24, 26]–which reinforces the “gender penalty” [15] in which women fall to the bottom of the occupational and economic hierarchies. The literature in the mapping describes midwifery as professionally undervalued with a subsequent lack of economic investment due to its socio-cultural feminisation. This dynamic can result in burn out and moral distress, as well as poor quality of care for women and newborns. There could be potential, not established through this mapping, for the barriers detailed here to lead to the mistreatment of women during childbirth.

The issues of social discrimination, work place hierarchy and power structures, lack of safety, basic remuneration and limited leisure time for providers of midwifery care, highlighted throughout the literature, place this issue urgently on the human rights agenda and beyond that of health system management only. We need to better differentiate the impact of barriers faced by midwifery personnel working in hospital facilities and those based in in communities. More needs to be understood about the impact of gender inequality on the provision of quality of care in relation to all three barriers. We need to find out what interventions exist to overcome the barriers and improve quality care.

The new Global Strategy for Women’s, Children’s and Adolescents Health (2016–2030) [17] builds upon guiding principles that include a gender responsive, equity driven and human rights based approach. We conclude, in light of the findings from the literature and in support of the new Global Strategy, that there is an urgent need for on-going mechanisms to improve quality of care to address the barriers as experienced by providers of midwifery care, as well as the gender inequality and rights issues that underlie them.

## Supporting Information

S1 Table Table of Search terms. (DOCX)

S2 Table Table of Included items. (DOCX)
