# Supplementary material for: What Prevents Quality Midwifery Care? A Systematic Mapping of Barriers in Low and Middle Income Countries from the Provider Perspective
Source: PLoS One. 2016 May 2;11(5):e0153391. doi: 10.1371/journal.pone.0153391 (PMC4852911; doi:10.1371/journal.pone.0153391)
Supplement: S1 Table — (DOCX) [file pone.0153391.s001.docx]

S1 Table of Search Terms

This table provides the search terms that were individually adapted to each electronic database. It is not registered with Prospero.

**Search terms and their combinations**

| **Provider Terms** | **Barrier Terms** | **Low and Middle Income Countries Filter** |
| --- | --- | --- |
| ("midwife" OR "midwives" OR "nurse midwife" OR "skilled birth attendant*" OR "Midwifery” OR "Nurse Midwives" OR "Maternal-Child Nursing") | "professional practice" OR “socioeconomic factors” OR "professional licenses" OR "professional identity" OR "professional associations" OR "professional education of women" OR "leadership" OR "Employee empowerment" OR "Accreditation (Education)" OR "Professional practice" OR "Multidisciplinary practices" OR "Professional relationships" OR "organisational structure" OR "Prejudices" OR "Sex Discrimination" OR "discrimination" OR "communication barriers" OR "gender inequality" OR "sexism" OR "gender identity" OR "social status" OR "social context" OR "social marginality" OR "conformity" OR "Social Values" OR "Social Perception" OR "Social Change" OR "social justice" OR "Femininity" OR "Stereotyping" OR "Women's Rights" OR "Feminism" OR "Masculinity" OR "evidence based nursing" OR "evidence based education" OR "Community Health Services" OR "Health Services Accessibility" OR "health services administration" OR "rural caregivers" OR "rural health services" OR "[Education](http://javascript:XslPostBack('ctl00$ctl00$MainContentArea$MainContentArea$xslResults','ThesaurusLink','LinkTarget%7CauthorityDetail%24LinkTerm%7CSU%2B%2522MIDWIVES%2B%2D%2D%2BEducation%2522%24ResultID%7C5');)" OR "Legal status” OR “Supervision" OR "Career development" OR "Government Policy" OR "public policy" OR "legislation" OR "finance" OR "grants" OR "economics" OR "government regulation" OR "regulat*" or "Opinion" OR "Point of view" OR "voice" OR "Questionnaires" OR "working environment" OR "working hours” OR "training" OR "quality service" OR "quality of life" OR "attitude” OR “psychology" OR "Twinning" OR "twin project" OR "autonomy" OR "association*" OR "empower*" OR "Advocacy" OR "strength*" OR "mentor" OR "Power" OR "barrier*" OR "prejudice*" OR "sexual discrimination" or "challenge*" OR "obstacle*" OR "hierarchy" OR "social status" OR "Women's status" OR "Women, Working" OR "Sexism" OR "Conformity" OR "Marginalization" OR "Sexual abuse" OR "abuse" OR "Skills" OR "rural" OR "Accountability" OR "legislation" OR "Mandatory Reporting" OR "liability" OR "funding" or "Opinion" OR "Point of view" OR "voice" OR "viewpoint" OR "Disillusion*" OR "hardship" OR "burn out" OR "Burnt out" OR "accommodation" OR "transport" OR "support" OR "Isolat*" or "Stress" OR "guilt" OR "Reality" OR "lived experience" OR "24 hour shift*" OR "pay" OR "salar* OR "danger" OR "overworked" OR "safe" OR "exhausted" OR "demotivat*” OR "Working hours" OR "trained" OR "Quality of Health Care" OR "quality improvement" OR "ethics" OR "register" OR "scope of practice" OR "code of conduct" OR "role" OR "practice development" OR "update" OR "Quality of Care" OR "Motivation" OR "Job Satisfaction" OR "working conditions" OR "security" OR "personal safety" OR "quality of life" OR "living conditions" OR "enabling environment" ) | Low and Middle Income Countries Filter |
