# Supplementary material for: What Prevents Quality Midwifery Care? A Systematic Mapping of Barriers in Low and Middle Income Countries from the Provider Perspective
Source: PLoS One. 2016 May 2;11(5):e0153391. doi: 10.1371/journal.pone.0153391 (PMC4852911; doi:10.1371/journal.pone.0153391)
Supplement: S2 Table — (DOCX) [file pone.0153391.s002.docx]

S2 Table of Included Items

| **N** | **Reference** | **Country/ries** | **Literature/ Study type** | **Provider title** | **Category of Barrier** |
| --- | --- | --- | --- | --- | --- |
| 1 | WHO (2000), Nursing and midwifery services facing crisis, experts say. Note for the press, No. 17, Geneva: World Health Organization. | Global | Press Release | Nurses and midwives | Professional/ social/ economic |
| 2 | de Bernis L, Sherratt DR, AbouZahr C & Van Lerbergh W. Skilled attendants for pregnancy, childbirth and postnatal care. British Medical bulletin. 2003; 67; 39-67. | LMICs | International Agency report | SBAs | Professional/ social |
| 3 | Kwast BE & Bentley J. Introducing confident midwives: midwifery education - action for safe motherhood. Midwifery. 1991; 7; 1; 8-19. | LMICs | Journal article, no empirical data | Midwives | Professional/  economic |
| 4 | Kwast BE. Midwives: key rural health workers in maternity care. International journal of gynaecology and obstetrics supplement. 1992; 38; S09-S15. | LMICs | Journal article, no empirical data | Midwives | Professional/  economic |
| 5 | Martinez AM, Khanh Khu DT, Boo NY, Neou L, Saysanasongkham B & Partridge JC. Barriers to neonatal care in developing countries: parents' and providers' perceptions. Journal of Paediatric Child Health. 2012; 48; 9; 852-858. | LMICs | Descriptive Study | Neonatal Healthcare providers: physicians; midwives; nurses; pediatric and nursing trainees) | Professional/ social |
| 6 | Peters MH. Midwives and the achievement of safer motherhood. International Journal of Gynecology and Obstetrics. 1995; 50; Supplement 2; S89-S92 | LMICs | Journal article, no empirical data | Midwives | Professional/ social/ economic |
| 7 | Riley,PL, Anderson B, Noguchi L, & Vindigni SM. Caring for global caregivers: a call to action. Journal of midwifery & women's health. 2005. 50 (4):  265-268. | LMICs | Journal article, no empirical data | Midwives | Professional/  economic |
| 8 | Riley PL. Advancing a midwifery perspective in health systems and human resources. African J Midwifery and Women's Health. 2011; 4; 5; 162.  <http://dx.doi.org/10.12968/ajmw.2011.5.4.162> | LMICs | Guest Editorial | Midwifery practitioners | Professional |
| 9 | Save the Children. Women on the Front Lines of Health Care. State of the World's Mothers 2010, Save the Children. 2010. | LMICs | International Agency report | Female health workers | Social |
| 10 | UNFPA-ICM. Investing in midwives and others with midwifery skills to save the lives of mothers and newborns and improve their health. New York, USA, United Nations Population Fund (UNFPA). 2007. | LMICs | International Agency report | Midwives and SBAs | Professional |
| 11 | Thomson A. The joint WHO/ICM/FIGO statement on skilled attendants at birth. Midwifery. 2005; 21; 1. | LMICs | International Agency statement | SBAs | Professional/ social/ economic |
| 12 | Trevisanuto D, Bavuusuren B, Wickramasinghe CS, Dharmaratne SM, Doglioni N, Giordan A, Zanardo V, & Carlo AW. Improving maternal and neonatal departments in high and low resource settings: the opinion of local health providers. Journal of Maternal-Fetal and Neonatal Medicine, 2011; 24(10): 1267-1272. | LMICs | Descriptive study | Maternal and neonatal nurses, midwives and physicians | Professional/  economic |
| 13 | Fullerton JT, Johnson PG, Thompson JB, Vivio D. Quality considerations in midwifery pre-service education: Exemplars from Africa. Midwifery. 2011 Jun;27(3):308-15. doi: 10.1016/j.midw.2010.10.011 | Africa | Descriptive study | Midwives, student midwives, donors, policy makers | Professional |
| 14 | Pettersson KO. Major challenges of midwifery in Africa. British Journal of Midwifery. 2007; 15; 8; 470-474. | Africa | Journal article, no empirical data | Midwives | Professional/ social/economic |
| 15 | Belizan M, Meier A, Althabe F, Agustina Codazzi A, Colomar M, Buekens P et al. Facilitators and barriers to adoption of evidence-based perinatal care in Latin American hospitals: a qualitative study. Health Education Research. 2007; 22; 6; 839-853. | Latin America | Descriptive study | Maternal health providers : midwives; obstetricians and gynaecologists | Professional |
| 16 | Bogren M U, [Wiseman A](http://www.ncbi.nlm.nih.gov/pubmed/?term=Wiseman%20A%5BAuthor%5D&cauthor=true&cauthor_uid=22578753) & [Berg M](http://www.ncbi.nlm.nih.gov/pubmed/?term=Berg%20M%5BAuthor%5D&cauthor=true&cauthor_uid=22578753). Midwifery education, regulation and association in six South Asian countries--a descriptive report. Sex Reprod Healthc. 2012 Jun;3(2):67-72. | South Asia | Descriptive study | Midwives | Professional |
| 17 | Utz B. [Siddiqui G](http://www.ncbi.nlm.nih.gov/pubmed/?term=Siddiqui%20G%5BAuthor%5D&cauthor=true&cauthor_uid=23656549), [Adegoke A](http://www.ncbi.nlm.nih.gov/pubmed/?term=Adegoke%20A%5BAuthor%5D&cauthor=true&cauthor_uid=23656549), [van den Broek N](http://www.ncbi.nlm.nih.gov/pubmed/?term=van%20den%20Broek%20N%5BAuthor%5D&cauthor=true&cauthor_uid=23656549).; Definitions and roles of a skilled birth attendant: a mapping exercise from four South-Asian countries. 2013. Acta Obstet Gynecol Scand. 92(9):1063-9. | South Asia | Descriptive study | SBAs | Professional |
| 18 | Martis R, Ho J J & Crowther C A. Survey of knowledge and perception on the access to evidence-based practice and clinical practice change among maternal and infant health practitioners in South East Asia. 2008. BMC Pregnancy & Childbirth, 8, 1-10 | South East Asia | Descriptive Study | Maternal Health and infant healthcare providers | Professional |
| 19 | Craig S. Working at the CURE Hospital in Kabul, Afghanistan. 2006. Midwifery Today Int Midwife. Spring. (77):44. | Afghanistan | Published Letter | Midwives | Professional/ social/economic |
| 20 | Currie S, Azfar P & Fowler RC. A bold new beginning for midwifery in Afghanistan Midwifery.2007; 23; 226–234. | Afghanistan | Journal article, no empirical data | Midwives | Social/ Economic |
| 21 | Rahmani Z & Brekke M. Antenatal and obstetric care in Afghanistan -a qualitative study among health care receivers and health care providers. BMC Health Services Research. 2013; 13; 166-175 | Afghanistan | Descriptive study | Midwives, doctors. | Professional/ social/economic |
| 22 | Turkmani S, Currie S, Mungia J, Assefi N, Rahmanzai A J, Azfar P & Bartlett L. Midwives are the backbone of our health system: Lessons from Afghanistan to guide expansion of midwifery in challenging settings. Midwifery. 2013; 29; 10; 1166-1172. | Afghanistan | Descriptive study | Midwives | Professional/ social/economic |
| 23 | Wood ME, Farooq Mansoor G, Hashemy P, Namey E, Fatima Gohar F, Fayeq Ayoubi S & Todd CS. Factors influencing the retention of midwives in the public sector in Afghanistan: A qualitative assessment of midwives in eight provinces. Midwifery.2013; 29; 10; 1137-1144. | Afghanistan | Descriptive study | Midwives, midwifery students, Community Health Workers, Health facility manager. | Professional/ social/economic |
| 24 | Pettersson KO, Svensson ML & Christensson K. The lived experiences of autonomous Angolan midwives working in midwifery-led maternity units. Midwifery. 2001; 17; 102-114. | Angola | Descriptive study | Midwives | Professional/ social/economic |
| 25 | Blum LS, Sharmin T & Ronsmans C. Attending home vs. clinic-based deliveries: perspectives of skilled birth attendants in Matlab, Bangladesh. Reproductive Health Matters. 2006; 14; 27; 51-60. | Bangladesh | Descriptive study | SBAs | Professional/  social |
| 26 | Narchi N Z, Pereira da Silva L C F, Gualda D M R & Bastos M H. Reclaiming direct-entry midwifery training in Brazil:context, challenges and perspectives. 2010*.* Midwifery 26, 385-388 | Brazil | Commentary | Midwives | Professional |
| 27 | Narchi N Z. Exercise of essential competencies for midwifery care by nurses in Sao Paulo, Brazil. Midwifery. 2011; 27; 1; 23-9. | Brazil | Descriptive study | Maternity nurses and midwives | Professional |
| 28 | Prytherch H, Moubassira Kagoné M, Aninanya GA, Williams JE, Kakoko DCV, Leshabari MT et al. Motivation and incentives of rural maternal and neonatal health care providers: a comparison of qualitative findings from Burkina Faso, Ghana and Tanzania. BMC Health Services Research. 2013; 13; 149-164. | Burkina Faso, Ghana and Tanzania. | Descriptive study | Maternal and neonatal health care providers | Professional/ social/economic |
| 29 | Cheung NG, Liping Zhang L, Mander R, Xu X & Wang X. Proposed continuing professional education programme for midwives in China: New mothers' and midwives' views. Nurse Education Today. 2011; 31; 434–438. | China | Descriptive study | Midwives | Professional |
| 30 | Mander R. The politics of maternity care and maternal health in China. 2010. Midwifery. 26(6):569-72. | China | Commentary | Midwives and nurses | Social |
| 31 | Tapley D. A life less ordinary: a British midwife in Ethiopia, Part 1. Marshalling the troops. 2009. Practising Midwife, 12(1), 24-26 | Ethiopia | Journal article, no empirical data | midwives and nurse/midwives | Professional |
| 32 | Floyd L. Helping midwives in Ghana to reduce maternal mortality. African Journal of Midwifery and Women's Health. 2013; 7; 1; 34-38. | Ghana | Descriptive study | Midwives | Professional |
| 33 | Lori JR, Rominski SD, Gyakobo M, Muriu EW, Kweku NE & Agyei-Baffour P. Perceived barriers and motivating factors influencing student midwives' acceptance of rural postings in Ghana. Human Resources for Health. 2012; 10; 17; doi:10.1186/1478-4491-10-17 | Ghana | Descriptive study | Student midwives | Professional/  social |
| 34 | Lori JR, Rominski SD, Richardson J, Gyakobo M, Muriu EW, Kweku NE et al. Factors influencing Ghanaian midwifery students’ willingness to work in rural areas: A computerized survey. International Journal of Nursing Studies. 2012; 49; 834-841. | Ghana | Descriptive study | Midwifery students | Professional/ social/economic |
| 35 | Moyer C, Akawire Aborigo R, Logonia G, Affah G, Rominski S, Adongo PB et al. Clean delivery practices in rural northern Ghana: a qualitative study of community and provider knowledge, attitudes, and beliefs. BMC Pregnancy Childbirth. 2012; 12; 50. doi: 10.1186/1471-2393-12-50 | Ghana | Descriptive study | TBAs and SBAs | Social |
| 36 | Hussein J, Phoya A, Ansong Tornui J & Okiwelu T. Midwifery practice in Ghana and Malawi: influences of the health system In: Reid L. editor. Freedom to practise: An international exploration of midwifery practice. Churchill Livingstone Elsevier: London; 2007. pp. 75-99. | Ghana and Malawi | Descriptive study | Midwives | Professional/  social |
| 37 | Mavalankar D, [Sankara Raman P](http://www.ncbi.nlm.nih.gov/pubmed/?term=Sankara%20Raman%20P%5BAuthor%5D&cauthor=true&cauthor_uid=20708311) & [Vora K](http://www.ncbi.nlm.nih.gov/pubmed/?term=Vora%20K%5BAuthor%5D&cauthor=true&cauthor_uid=20708311). Midwives of India: missing in action. 2011. Midwifery. 2011; 27; 5; 700-6. | India | Journal article, no empirical data | Midwives | Professional/ social/ economic |
| 38 | Prasad R & [Dasgupta](http://www.ijcm.org.in/searchresult.asp?search=&author=Rajib+Dasgupta&journal=Y&but_search=Search&entries=10&pg=1&s=0) R. Missing midwifery: relevance for contemporary challenges in maternal health. Indian Journal of Community Medicine. 2013; 38; 1; 9-14. | India | Journal article, no empirical data | SBAs and auxiliary nurse-midwives | Professional |
| 39 | Sharma B, Johansson E, Prakasamma M, Mavalankar M & Christensson K. Midwifery scope of practice among staff nurses: A grounded theory study in Gujarat, India. Midwifery. 2013; 29; 628-636. | India | Descriptive study | Maternity staff members | Professional |
| 40 | Burke L, [Suswardany DL](http://www.ncbi.nlm.nih.gov/pubmed/?term=Suswardany%20DL%5BAuthor%5D&cauthor=true&cauthor_uid=21410993), [Michener K](http://www.ncbi.nlm.nih.gov/pubmed/?term=Michener%20K%5BAuthor%5D&cauthor=true&cauthor_uid=21410993), [Mazurki S](http://www.ncbi.nlm.nih.gov/pubmed/?term=Mazurki%20S%5BAuthor%5D&cauthor=true&cauthor_uid=21410993), [Adair T](http://www.ncbi.nlm.nih.gov/pubmed/?term=Adair%20T%5BAuthor%5D&cauthor=true&cauthor_uid=21410993), [Elmiyati C](http://www.ncbi.nlm.nih.gov/pubmed/?term=Elmiyati%20C%5BAuthor%5D&cauthor=true&cauthor_uid=21410993) et al. Utility of local health registers in measuring perinatal mortality: A case study in rural Indonesia. BMC Pregnancy Childbirth. 2011; 17; 11-20. | Indonesia | Descriptive study | Midwives | Professional |
| 41 | D'Ambruoso L, Achadi E, Adisasmita A, Izati Y, Makowiecka K & Hussein J. Assessing quality of care provided by Indonesian village midwives with a confidential enquiry*.* Midwifery. 2009; 5; 25; 528-539. | Indonesia | Descriptive study | Health providers, family and communities | Professional/  social |
| 42 | D'Ambruoso L, Byass P & Qomariyah ST. Final Caregivers' Perspectives on access to emergency obstetric care in Indonesia. Journal of Biosocial Science. 2010; 42; 2; 213-241. | Indonesia | Descriptive study | Maternal health providers | Professional/ social/economic |
| 43 | Ensor T, Quayyum Z, Nadjib M & Sucahya P. Level and determinants of incentives for village midwives in Indonesia. Health Policy and Planning. 2009; 24; 26-35. | Indonesia | Descriptive study | Midwives | Social |
| 44 | Makowiecka K, Achadi E, Izati Y & Ronsmans C. Midwifery provision in two districts in Indonesia: how well are rural areas served? Health Policy Plan. 2008; 23; 1; 67-75. | Indonesia | Descriptive study | Midwives | Professional/ social/economic |
| 45 | Shankar A, Sebayang S, Guarenti L, Utomo B, Islam M, Fauveau V & Jalal F. The village-based midwife programme in Indonesia. The Lancet. 2008; 371; 9620; 1226-1229. | Indonesia | Commentary | Midwives | Professional/  economic |
| 46 | Mohammad-Alizadeh CS, Wahlstrom R, Vahidi R, Nikniaz A, Marions L & Johansson A. Barriers to high-quality primary reproductive health services in an urban area of Iran: views of public health providers. Midwifery. 2009; 25; 721–730. | Iran | Descriptive study | Midwives and maternity health providers. | Professional/ social/economic |
| 47 | Rankin S. Climbing walls and leaping fences: women's issues in west bank and Gaza strip. BJM. 1996; 4: 12; 660- 661. | Israel | Journal article, no empirical data | Midwives | Professional/ social/economic |
| 48 | Shaban I, Barclay L, Lock L & Homer C. Barriers to developing midwifery as a primary health-care strategy: A Jordanian study. Midwifery. 2012; 28; 106-111. | Jordan | Descriptive study | Midwives and midwifery educators | Professional/  social |
| 49 | Turan JM, Bukusi EA, Cohen CR, Sande J & Miller S. Effects of HIV/AIDS on Maternity Care Providers in Kenya. Journal of Obstetric, Gynecologic, & Neonatal Nursing. 2008; 37; 5; 588-595. | Kenya | Descriptive study | Nurse -midwives, physicians, physician assistants | Professional/ social/economic |
| 50 | Beltman JJ, van den Akker T, Bwirire D, Korevaar A, Chidakwani R, Lonkhuijzen L et al. Local health workers' perceptions of substandard care in the management of obstetric hemorrhage in rural Malawi. BMC Pregnancy Childbirth. 2013; 13; 39-44. | Malawi | Descriptive study | nurse-midwives and non physician clinicians | Professional |
| 51 | Bream KDW, Gennaro S, Kafulafula U, Mbweza E & Hehir D. Barriers to and facilitators for newborn resuscitation in Malawi, Africa. Journal of Midwifery and Women's Health. 2005; 50; 4; 329-334. | Malawi | Descriptive study | Nurses/nurse midwives | Professional |
| 52 | Thorsen VC, Teten Tharp AL, & Meguid T. High rates of burnout among maternal health staff at a referral hospital in Malawi. BMC Nursing. 2011; 23: 9; doi:10.1186/1472-6955-10-9. | Malawi | Descriptive study | Maternal health staff | Professional/  social |
| 53 | Hurley EA, Warren NE, Doumbia S & Winch PJ. Exploring the connectedness of rural auxiliary midwives to social networks in Koutiala, Mali. Midwifery. 2013; 30; 1; 123-129. | Mali | Descriptive study | Midwives | Professional/  economic |
| 54 | Kildea S. Review of midwifery in Mongolia utilising the 'Strengthening Midwifery Toolkit'. Women Birth. 2012; 25; 4; 166-173. | Mongolia | Descriptive study | Midwives and student midwives | Professional/ social/economic |
| 55 | Kerouac S. Strengthening midwifery practices in Morocco: a gender perspective. International Midwifery. 2005; 18; 1; 8-9. | Morocco | Journal article, no empirical data | Midwives | Professional/ social |
| 56 | Temmar F, [Vissandjée B](http://www.ncbi.nlm.nih.gov/pubmed/?term=Vissandj%C3%A9e%20B%5BAuthor%5D&cauthor=true&cauthor_uid=16713882), [Hatem M](http://www.ncbi.nlm.nih.gov/pubmed/?term=Hatem%20M%5BAuthor%5D&cauthor=true&cauthor_uid=16713882), [Apale A](http://www.ncbi.nlm.nih.gov/pubmed/?term=Apale%20A%5BAuthor%5D&cauthor=true&cauthor_uid=16713882), & [Kobluk D](http://www.ncbi.nlm.nih.gov/pubmed/?term=Kobluk%20D%5BAuthor%5D&cauthor=true&cauthor_uid=16713882). Midwives in Morocco: seeking recognition as skilled partners in women-centred maternity care. Reprod Health Matters. 2006; 14; 27; 83-90. | Morocco | Journal article, no empirical data | Midwives | Professional/ social/economic |
| 57 | Pettersson KO, Eva Johansson E, de Fatima M, Pelembe M, Dgedge C, & Christensson K. Mozambican midwives' views on barriers to quality perinatal care. Health Care Women International. 2006; 27; 2; 145-168. | Mozambique | Descriptive study | Midwives | Professional/  social |
| 58 | Carlough M. & McCall M. Skilled birth attendance: What does it mean and how can it be measured? A clinical skills assessment of maternal and child health workers in Nepal. International Journal of Gynecology and Obstetrics. 2005; 89; 200—208. | Nepal | Descriptive study | Maternal and child health workers | Professional/  social |
| 59 | Chhetry S, Clapham S & Basnett I. Community based maternal and child health care in Nepal: self-reported performance of Maternal and Child Health Workers. Journal of Nepal Medical Association. 2005; 44; 157; 1-7. | Nepal | Descriptive study | Maternal and Child Health Workers | Professional/ social/economic |
| 60 | Clapham S, Pokharel D, Bird C, & Basnet I. Addressing the attitudes of service providers: increasing access to professional midwifery care in Nepal. Tropical Doctor,. 2008; 38; 4; 197-201. | Nepal | Journal article, no empirical data | Midwifery service providers | Professional/  social |
| 61 | Kc A & Bajracharya K. State of midwives in Nepal: HRH to improve maternal and neonatal health and survival. Journal of Nepal Health Research Council. 2013; 11; 23; 98-101. | Nepal | Journal article, no empirical data | Midwives | Professional/ social/economic |
| 62 | Jaffre Y & Prual A. Midwives in Niger: an uncomfortable position between social behaviours and health care constraints. Social Science Medicine. 1994; 38; 8; 1069-1073. | Niger | Descriptive study | Midwives | Social |
| 63 | Abimbola S, Okoli U, Olubajo O, Abdullahi MJ & Pate MA. The Midwives Service Scheme in Nigeria. PLoS Med. 2012; 9; 5. doi:10.1371/journal.pmed.1001211 | Nigeria | Intervention Study on impact of the midwives service scheme | Midwives | Professional/ social/economic |
| 64 | Fauveau V, Sherratt DR & de Bernis L. Human resources for maternal health: multi-purpose or specialists? Human Resources for Health. 2008; 6; 21. doi:10.1186/1478-4491-6-21 | Nigeria | Journal article, no empirical data | Maternal health providers | Professional/  social |
| 65 | Ezeonwu MC. Maternal birth outcomes: processes and challenges in Anambra State, Nigeria. Health Care Women International. 2011; 32; 6; 492-514. | Nigeria | Descriptive study | Nursing and midwifery educators; hospital administrators; Nursing and Midwifery Council of Nigeria members. | Professional/ social/economic |
| 66 | Gibson H. Training midwives in inner-city Karachi, Pakistan. British Journal of Midwifery. 2000; 8; 6; 374-378. | Pakistan | Journal article, no empirical data | Midwives | Professional/ social/economic |
| 67 | Huicho L, Miranda JJ, Diez-Canseco F, Lema C, Lescano AG, Lagarde M & Blaauw D. Job Preferences of Nurses and Midwives for Taking Up a Rural Job in Peru: A Discrete Choice Experiment. PLoS ONE. 2012; 7; 12: e50315. doi:10.1371/journal.pone.0050315 | Peru | Descriptive study | Nurses and midwives | Professional/ social/economic |
| 68 | de la Gente A. Midwifery in the Philippines: 'a laudable service' but there are issues and challenges. International Midwifery. 2008; 4; 21-23 | Philippines | Journal article, no empirical data | Midwives | Professional |
| 69 | Roxburgh M. Rwanda: A thousand hills, a thousand dreams, a thousand challenges for nurses and midwives and the Millennium Development Goals. Nurse Education in Practice. 2009; 9, 349-350. | Rwanda | Editorial | Nurses and midwives | Professional |
| 70 | Rouleau D, Fournier P, Philibert A, Mbengue B, & Dumont A. The effects of midwives’ job satisfaction on burnout, intention to quit and turnover: a longitudinal study in Senegal. Human Resources for Health. 2012; 10; 9; doi:  [10.1186/1478-4491-10-9](http://dx.doi.org/10.1186%2F1478-4491-10-9). | Senegal | Descriptive study | Midwives | Professional/ economic |
| 71 | Nyango DD, Mutihir T, Laabes EP, Kigbu JH & Buba M. Skilled Attendance: The Key Challenges to Progress in Achieving MDG 5 in North Central Nigeria. African Journal of Reproductive Health. 2010; 14; 2; 130-138. | Sierra Leone | Descriptive study | Nurse-midwives | Professional/  economic |
| 72 | Khalil S., Larsson M. & Govind, S. Violence against midwives in Cape Town. African Journal of Midwifery and Women's Health. 2009; 3; 1; 37-40. | South Africa | Descriptive study | midwives | Professional/  social |
| 73 | Schoon GM. Discussion platform between midwifery educators and health professionals in the Free State province. South African Journal of Obstetrics and Gynaecology. 2011; 17; 2; 28-30. | South Africa | Journal article, no empirical data | Midwifery educators and health professionals | Professional |
| 74 | Schoon GM & Motlolometsi MWA. Poor maternal outcomes: a factor of poor professional systems design. South African Medical Journal. 2012; 102; 10; 784-786. | South Africa | Commentary | Maternity nurses, midwives and maternity staff | Professional |
| 75 | Taylor LC, Fair CD & Nikodem C. Working conditions and perspectives among South African health workers. African Journal of Midwifery and Women's Health. 2011; 5; 4; 176-180. | South Africa | Descriptive study | Midwives, nurses and nursing students. | Professional |
| 76 | Khalfaoui, M, [Njah M](http://www.ncbi.nlm.nih.gov/pubmed/?term=Njah%20M%5BAuthor%5D&cauthor=true&cauthor_uid=11084469) & [Zoghlami H](http://www.ncbi.nlm.nih.gov/pubmed/?term=Zoghlami%20H%5BAuthor%5D&cauthor=true&cauthor_uid=11084469). Conditions of midwifery practice in Tunisia rural areas and maternal transfer.2000. J Gynecol Obstet Biol Reprod. 2000; 29; 6; 614-20. | Tunisia | Descriptive study | Midwives | Professional/ social |
| 77 | Sogukpinar N, [Saydam BK](http://www.ncbi.nlm.nih.gov/pubmed/?term=Saydam%20BK%5BAuthor%5D&cauthor=true&cauthor_uid=17499895), [Bozkurt OD](http://www.ncbi.nlm.nih.gov/pubmed/?term=Bozkurt%20OD%5BAuthor%5D&cauthor=true&cauthor_uid=17499895), [Ozturk H](http://www.ncbi.nlm.nih.gov/pubmed/?term=Ozturk%20H%5BAuthor%5D&cauthor=true&cauthor_uid=17499895) & [Pelik A](http://www.ncbi.nlm.nih.gov/pubmed/?term=Pelik%20A%5BAuthor%5D&cauthor=true&cauthor_uid=17499895). Past and present midwifery education in Turkey. Midwifery. 2007; 23; 4; 433-42. | Turkey | Journal article, no empirical data | Midwives | Professional |
| 78 | Carmel AS. Desirable, achievable, but not easy: The midwife role in Kalongo Pader. British Journal of Midwifery. 2006; 14; 5; 272-274. | Uganda | Journal article, no empirical data | Midwives | Professional |
| 79 | Ford L. Passionate Ugandan midwife takes her message abroad. The Guardian Newspaper. 12 Oct 2011. Available: <http://www.theguardian.com/global-development/poverty-matters/2011/oct/12/uganda-midwife-maternal-mortality-campaign>. | Uganda | News article | Midwives | Professional |
| 80 | Franngard C, Hansevnden A, & Liljestrand J. Compassion and severe challenges- An exploratory study of being a midwife in rural Uganda. Midwifery Digest. 2006; 16; 4; 461-466. | Uganda | Descriptive study | Midwives | Professional/ social/economic |
| 81 | Kaye D. Quality of midwifery care in Soroti district, Uganda. East African Medical Journal. 2000; 77; 10; 558-561. | Uganda | Descriptive study | Midwives | Professional |
| 82 | Mudokwenyu-Rawdon C & Chaibva CN. Needs assessment and strengthening midwifery association in Zimbabwe. African Journal of Midwifery and Women's Health. 2010; 1; 4; 38-41. | Zimbabwe | Descriptive study | Midwives and midwifery association members | Professional/ social/economic |
